# Application of 16S rRNA gene amplicon sequencing in the investigation of novel ovine skin lesions in Norway

**DOI:** 10.3389/fvets.2026.1802983

**Published:** 2026-06-03

**Authors:** J. S. Duncan, J. W. Angell, L. Lenzi, X. Liu, G. J. Staton, N. J. Evans, M. Gilhuus

**Affiliations:** 1 Department of Livestock and One Health, Institute of Infection, Veterinary and Ecological Science, University of Liverpool, Wirral, United Kingdom; 2 Centre for Genomic Research, Department of Evolution, Ecology and Behaviour, Institute of Infection, Veterinary and Ecological Science, University of Liverpool, Liverpool, United Kingdom; 3 Department of Infection Biology & Microbiomes, Institute of Infection, Veterinary and Ecological Science, The University of Liverpool, Wirral, United Kingdom; 4 Animalia - Norwegian Meat and Poultry Research Centre, Oslo, Norway

**Keywords:** CODD, footrot, lameness, lesions, microbiome, sheep, skin

## Abstract

**Background:**

Contagious ovine digital dermatitis (CODD) is a globally emerging polymicrobial foot disease in sheep that causes severe welfare and economic problems. Currently, there is no validated commercial laboratory diagnostic test for CODD, and the current ‘gold standard’ is the pathological scoring of foot lesions. In this study, we used a combination of gross pathological lesion scoring and 16S rRNA gene amplicon sequencing of the bacterial microbiome to investigate novel suspected CODD foot lesions identified in three Norwegian abattoirs.

**Methods:**

We conducted 16S rRNA gene amplicon sequencing of biopsy samples from novel Norwegian foot lesions (*n* = 30), footrot lesions (*n* = 7), and healthy skin (*n* = 30), and the results were compared with sequenced biopsy samples from a previous study of sheep from the United Kingdom (UK) with CODD lesions (*n* = 31) and healthy foot skin (*n* = 7). A UK veterinarian with clinical experience in CODD performed the pathological scoring of the novel Norwegian foot lesions using photographic images collected at the Norwegian abattoirs.

**Results:**

Gross pathology and bacterial microbiome compositional analysis revealed that the novel CODD-like lesions were pathologically and bacteriologically distinct from healthy skin, CODD lesions, and footrot lesions. The novel CODD-like lesions presented as ulceration in the skin region immediately dorsal to the coronary band, extending distally below the carpus/tarsus, and were associated with hair loss, haemorrhage, and crusting. A comparison of the bacterial microbiota in the novel CODD-like lesions with those found in healthy skin, CODD lesions, and footrot lesions revealed that the bacterial communities were significantly different in terms of diversity, phylogeny, and microbial composition.

**Conclusion:**

Gross pathological lesion description, used in combination with 16S rRNA gene sequencing of the bacterial microbiome, demonstrated that the novel Norwegian skin lesions involved a dysbiosis that differed substantially from what has been previously described for CODD lesions and that the lesions were highly unlikely to be CODD. Further studies on the aetiopathogenesis of this novel sheep hoof condition should enable improved diagnosis.

## Introduction

Global livestock industries must contend with newly emerging and re-emerging animal diseases that can seriously affect animal welfare, economic stability, and the sustainability of these industries (1), as well as pose risks to human health (2). Recent instances in the sheep industry include the incursion of peste des petits ruminants into Europe (3) and the global outbreak of novel Schmallenberg orthobunyavirus infection (4). For many diseases, the clinical signs are not unique. Therefore, validated laboratory diagnostics for pathogen detection—such as bacterial culture to isolate causal pathogens, polymerase chain reaction (PCR) tests to detect bacterial genomic DNA, or enzyme-linked immunosorbent assay (ELISA) for identifying antigens or antibodies—are necessary to confirm the clinical diagnosis. For single-pathogen diseases, the development and validation of such assays can be relatively straightforward. However, for diseases of unknown aetiology or those involving a consortium of pathogens, the development of validated laboratory assays to support clinical diagnosis is much more challenging. A recent example of an emerging polymicrobial disease in sheep is contagious ovine digital dermatitis (CODD) (5).

CODD is a cause of severe lameness in sheep and was first identified in the UK in 1997 (6). Since then, the disease has become endemic in the UK (7) and has also been reported in Ireland (8), India (9), Iran (10), Germany (11), Sweden (12), and Switzerland (13). Due to the severity and contagious nature of the condition, veterinarians in countries that have not yet experienced CODD in their sheep flocks are concerned about its potential emergence.

**Figure 1:**
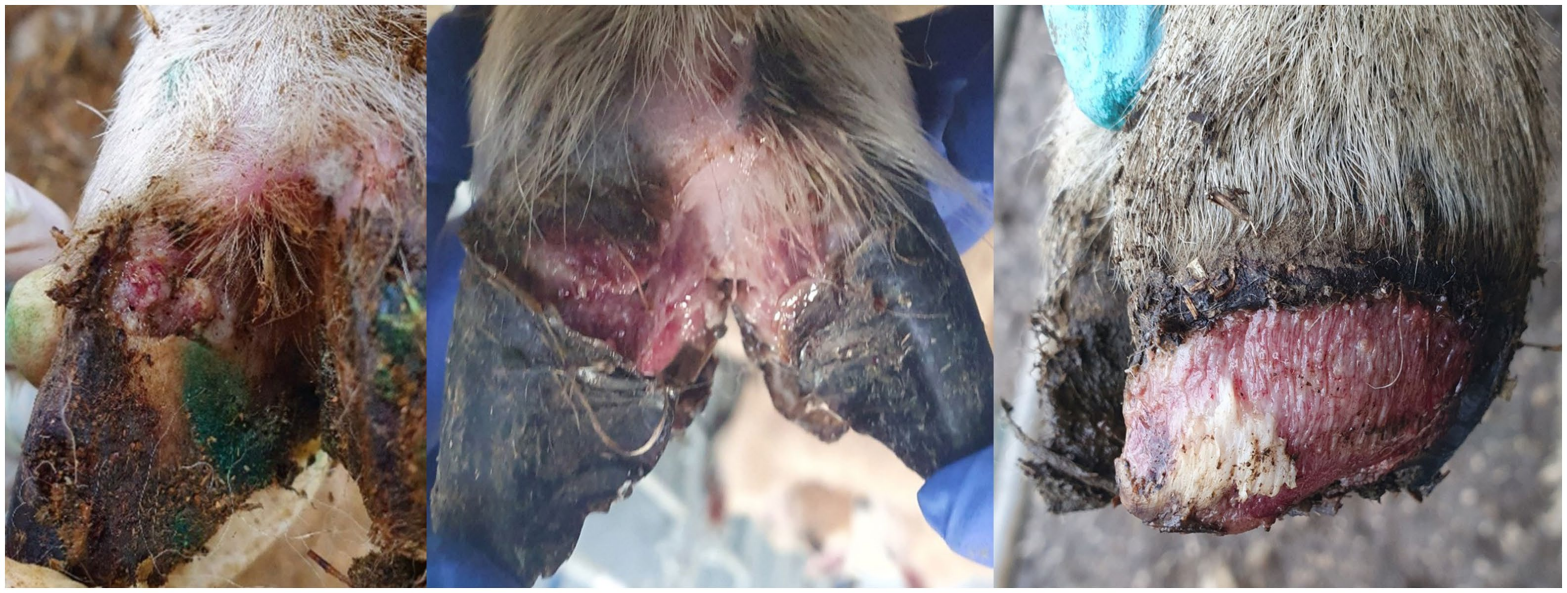
CODD lesions from UK sheep. Note that the lesions extend distal to the coronary band and are associated with separation of the hoof capsule from the underlying dermal laminae.

As the precise aetiology of CODD is still not fully understood, a validated laboratory microbiological diagnostic test is not yet available, and diagnosis still relies primarily on gross pathology using a recognised lesion scoring system (14). The condition begins with an initial inflammatory lesion at the coronary band of the hoof and progresses to the separation and detachment of the hoof capsule from the underlying sensitive laminae. This process continues until the hoof capsule eventually sloughs off completely (Figure 1) (14). Diagnosing CODD based on gross pathology requires experience with the condition and can be complicated by the presence of multiple disease processes in the foot, such as co-infection with benign or virulent footrot. In addition, other sheep foot diseases can cause similar lesions, such as foot abscesses, trauma (15), ulcerative skin disease (16), or orf virus infections (17).

Recent research has shown CODD to be polymicrobial, involving bacteria such as *Treponema pedis*, *Treponema medium*, *Treponema phagedenis*, *Dichelobacter nodosus* (the causative agent of footrot), and *Fusobacterium necrophorum* (a common pathogen of farm animals) (5). These anaerobic pathogens are difficult to isolate by conventional bacterial culture techniques and, therefore, this service is not generally available in veterinary diagnostic laboratories. Although PCR assays exist for all these pathogens, none have been validated as diagnostic tests for CODD in sheep (5, 18).

This study aims to determine whether the novel Norwegian lesions are indeed CODD by comparing their gross pathology and microbial profiles with a pre-existing UK CODD dataset (19). This comparison seeks to assess whether CODD may be emerging in Norway.

## Materials and methods

### Ethical approval

The collection of Norwegian biopsy tissue samples was approved by the Norwegian Food Safety Authority (FOTS ID 27336 and 30282) and subsequently approved by the University of Liverpool Animal Welfare Ethical Review Board (AWC0202). The collection of the UK biopsy samples was approved under the UK Animals (Scientific Procedures) Act (ASPA) 1986, Home Office Project Licence PPL 708756, and University of Liverpool Veterinary Ethics Committee approval number VREC417.

### Sample collection and foot lesion classification

Norwegian foot biopsy samples were collected by trained foot assessors (*n* = 2) as part of the Norwegian footrot surveillance programme at three abattoirs between 2020 and 2022. The assessors were provided with photographic sheets with standard scoring systems for CODD (14) and asked to photograph and collect biopsy tissue from any foot lesions that resembled CODD, as well as from the coronary band of healthy feet. The assessors wiped the skin or lesion with sterile cotton swabs to remove any surface contamination, and the biopsy samples were collected using a 6 mm sterile punch biopsy. The tissue biopsies were immediately transferred to tubes containing RNA*later*™ (Invitrogen, Thermo Fisher Scientific, Baltics, Vilnius, Lithuania). The samples were kept at 4 °C until they were transported to the laboratory, where they were stored at −20 °C until DNA extraction.

UK foot biopsy samples were collected and processed in 2016 from sheep on farms with a history of CODD. Sheep were examined by a veterinary surgeon, and the foot lesions were scored as CODD grades 1 to 5. Prior to biopsy sampling, all feet were cleaned with sterile dry cotton swabs to remove surface contamination. A biopsy was then obtained using a sterile 6 mm punch biopsy. The samples were transported back to the laboratory on ice and stored at −80 °C until DNA extraction.

### Extraction of DNA from Norwegian samples

DNA was extracted from the biopsies using the Invitrogen PureLink Microbiome Kit (Thermo Fisher Scientific, Carlsbad, CA, USA) following the manufacturer’s protocol with minor modifications. DNA quality and concentrations were assessed using the Multiskan Sky Microplate Spectrophotometer (Thermo Fisher Scientific) and the Tecan Spark fluorescence reader (Tecan, Männedorf, Switzerland) with the Qubit BR dsDNA kit (Thermo Fisher Scientific). A negative control (extraction reagents only) and a positive control [the ZymoBIOMICS Microbial Community Standard (Zymo Research Corporation, Irvine, CA, USA)] were included in the DNA extraction step.

### Extraction of DNA from UK samples

DNA was extracted using the DNeasy Blood and Tissue Kit (QIAGEN, Manchester, UK), as previously described in other 16S rRNA gene-targeted microbiota studies of ruminant digital dermatitis (20–22) according to manufacturers’ instructions. A negative (not containing any sample material) and a positive control [the ZymoBIOMICS Microbial Community Standard (Zymo Research Corporation, Irvine, CA, USA)] were included in the DNA extraction step. The extracted DNA samples were quantified using the Qubit BR dsDNA kit (Thermo Fisher Scientific) and were stored at −80 °C until use.

### 16S rRNA gene amplification and Illumina MiSeq sequencing

At the UK sequencing laboratory, DNA from samples in both locations was quantified using the Qubit BR dsDNA kit (Thermo Fisher Scientific) before submitting for *16S rRNA* gene V4 region amplification using a forward primer N501f and a reverse primer N701r, with adapter sequences to produce a 254 bp insert, as described previously (23). A negative control and a positive control (the ZymoBIOMICS Microbial Community DNA Standard (Cat. No. D6306)) were included. The libraries were sequenced on the Illumina^®^ MiSeq platform (Illumina^®^, San Diego, USA), generating 2 × 250 bp paired-end reads.

### Bioinformatic analysis

#### Sequencing read quality control and filtering

Base calling and demultiplexing of indexed reads were performed using CASAVA version 1.8.2 (Illumina) to produce sample sequence files in FASTQ format. The raw FASTQ files were then imported into the QIIME 22023.2 pipeline (24) and trimmed to remove Illumina adapter sequences and PCR primers using the QIIME 2 cutadapt trim-paired plugin [cutadapt version 1.2.1 (25, 26)].

#### Amplicon sequence variant (ASV) identification and taxonomy assignment

Amplicon sequence variants (ASVs) were generated using QIIME 2 (version 2022.8) and the q2-dada2 denoise-paired method [DADA2 version 1.26.0; (27), which corrects sequencing errors and infers exact biological sequences]. This step also produced a feature table of ASV abundances.

Following alignment of sequences using the multiple alignment using fast Fourier transform algorithm (MAFFT, version 7.515) and the QIIME 2 q2-alignment plugin (27), a phylogenetic tree was built and converted to a rooted format using the FastTree tool (28) via the QIIME 2 phylogeny fasttree plugin, with midpoint rooting applied.

Taxonomic classification of ASVs was performed using the q2-feature-classifier classify-sklearn method (scikit-learn version 0.24.1), with a confidence threshold of 0.7 and the SILVA database (version 138) as the reference.

Taxonomic profiles were visualised using the q2-taxa plugin. Feature tables were collapsed at multiple taxonomic levels and visualised as heatmaps using the q2-feature-table plugin.

### Diversity analysis

Alpha diversity analyses were performed using the QIIME 2 diversity core-metrics-phylogenetic plugin. The sequencing depth of all samples was explored using the Shannon index (28) plotted as a rarefaction curve. Following the normalisation of abundance counts by rarefaction at the selected threshold (70,000 sequences), the normalised counts were then used to compute alpha diversity using different metrics: Shannon diversity index (28), Simpson index (29), observed features, and Faith’s phylogenetic diversity (PD) (30). A Kruskal–Wallis test, followed by pairwise comparisons using Wilcoxon rank-sum tests and Benjamini–Hochberg correction for multiple testing, was carried out using the QIIME 2 diversity alpha-group-significance plugin. The significance threshold was set at 0.05.

Beta diversity among sample groups was investigated by performing normalisation by rarefaction using the QIIME 2 diversity core-metrics-phylogenetic plugin, followed by the Bray–Curtis dissimilarity (31), Jaccard index (32), and Weighted and Unweighted UniFrac dissimilarity measures (33, 34). These were produced using the QIIME 2 diversity core-metrics-phylogenetic plugin. PERMANOVA (35) was used to investigate the resulting distance matrices, using 999 permutations, with a *p*-value of < 0.05 set as the significance threshold.

### Analysis of differentially abundant ASVs/taxa among the sample groups

Differential abundance analysis was performed using the analysis of composition of microbiomes (ANCOM) method (36). The ANCOM method tests the hypothesis that the majority of ASVs do not differ in abundance between groups by comparing log-ratios of taxa across samples. For each pair of taxa (x and y), the method evaluates whether the log-ratio of their abundance differs between groups and counts the number of times the null hypothesis (H₀: taxon x is not differentially abundant) is rejected. This count is reported as the W statistic. The W statistic, therefore, represents the number of pairwise log-ratio tests in which the null hypothesis is rejected for a given taxon. Taxa were considered differentially abundant if the W value exceeded 70% of the total possible pairwise comparisons, corresponding to significance in the majority of tests.

Prior to analysis, ASVs were filtered to retain features present in at least three samples with a minimum total frequency of 10, in order to reduce the impact of sparsity and zero counts on ANCOM results.

Visualisations were conducted in R (37) using the packages biomformat (38), phyloseq (39), ggplot2 (40), and vegan (41). The data visualisation code was developed in R with assistance from ChatGPT (OpenAI). All outputs were reviewed, validated, and interpreted by the authors.

## Results

### Foot lesion classification

The Norwegian foot biopsy samples were collected for disease surveillance at abattoirs between 2020 and 2022. The examined feet (*n* = 67) were categorised as healthy, footrot, or CODD-like by the Norwegian samplers, based on established classification schemes (14, 42). Photographs of all lesions were subsequently scored by a UK veterinarian with substantial experience in CODD.

**Figure 2:**
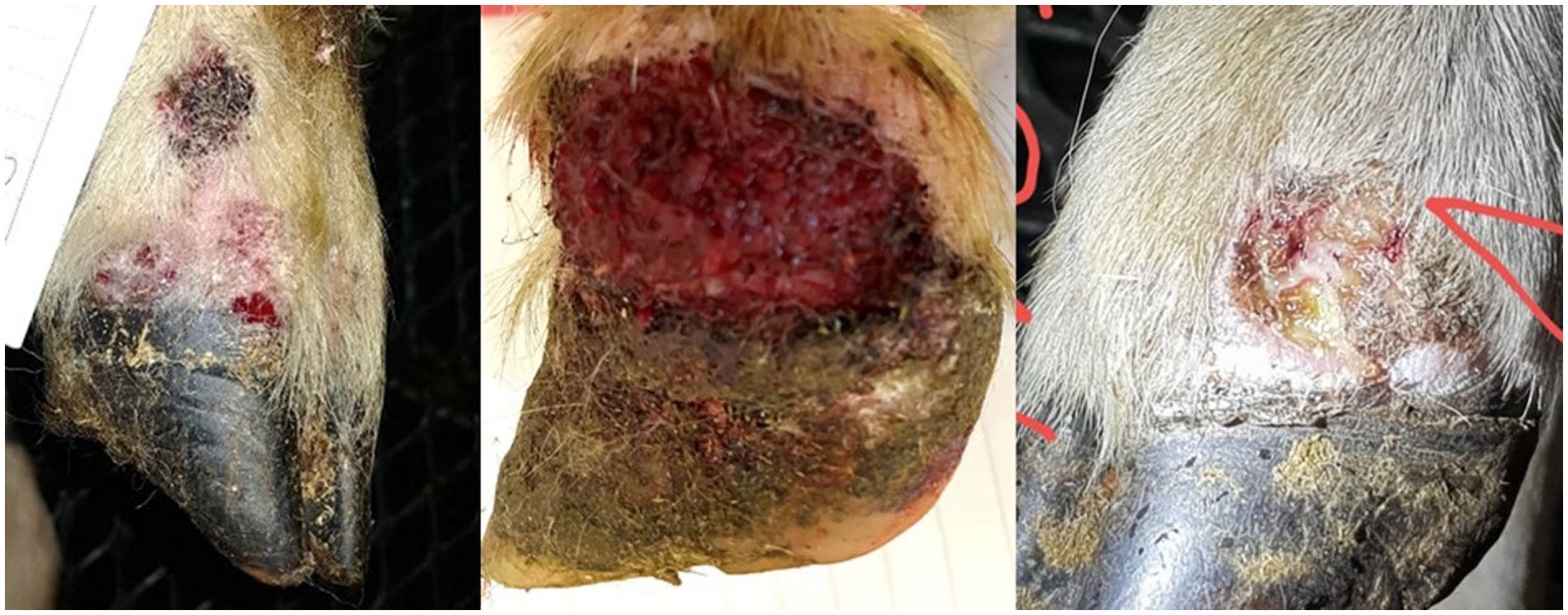
Examples of skin lesions classified as ‘CODD-like’, sampled at a Norwegian abattoir.

These CODD-like lesions all occurred in the skin region immediately dorsal to the coronary band, extending to below the fetlock (Figure 2). Lesion sizes ranged from approximately a few millimetres to several centimetres. Hair loss, evidence of haemorrhage, and crusting were present in all lesions. Some lesions had a proliferative appearance (*n* = 5). There was no evidence of separation of the hoof horn from the underlying sensitive laminae, which is characteristic of CODD lesions (Figure 1).

Hence, based on the gross pathological appearance of the foot lesion images, the clinical opinion of the UK veterinarian was that the 30 CODD-like lesions were not CODD. As a result, there were 30 CODD-like lesions, 7 mild benign footrot lesions, and 30 healthy foot samples in the Norwegian dataset.

The UK foot biopsy samples were collected from different UK farms in 2016, and foot lesions were classified as healthy (*n* = 7), active CODD (CODD grades 1–4) (*n* = 31), and healed CODD (Grade 5) (*n* = 8) by a UK veterinarian with experience in CODD using the same classification system.

### Sequencing results

A total of 113 sheep foot samples were included in the dataset. The median number of reads per sample was 116,932 (interquartile range, IQR: 74,295.5). A total of 1,863 different ASVs were identified and taxonomically assigned. All alpha and beta diversity analyses were performed at a sampling depth of 70,000 sequences using the QIIME 2 diversity core metrics-phylogenetic plugin. This value was obtained using the QIIME 2 diversity alpha rarefaction plugin and was selected to ensure maximum sampling depth while minimising the sample loss (108 samples; 95.57% retained). Inspection of the resulting alpha rarefaction curve was conducted to ensure adequate sequencing depth (Supplementary File 1). Consequently, the dataset comprised 66 Norwegian foot samples (CODD-like *n* = 30, benign footrot *n* = 7, and healthy *n* = 29) and 42 UK foot samples (CODD *n* = 28, healed CODD *n* = 8, and healthy *n* = 6) (Supplementary File 2).

### Microbiota comparisons of UK CODD and Norwegian CODD-like lesions

For this analysis, the microbiota of the 30 Norwegian CODD-like samples and 28 UK CODD samples (grades 1–4) were compared.

#### Alpha diversity

**Figure 3:**
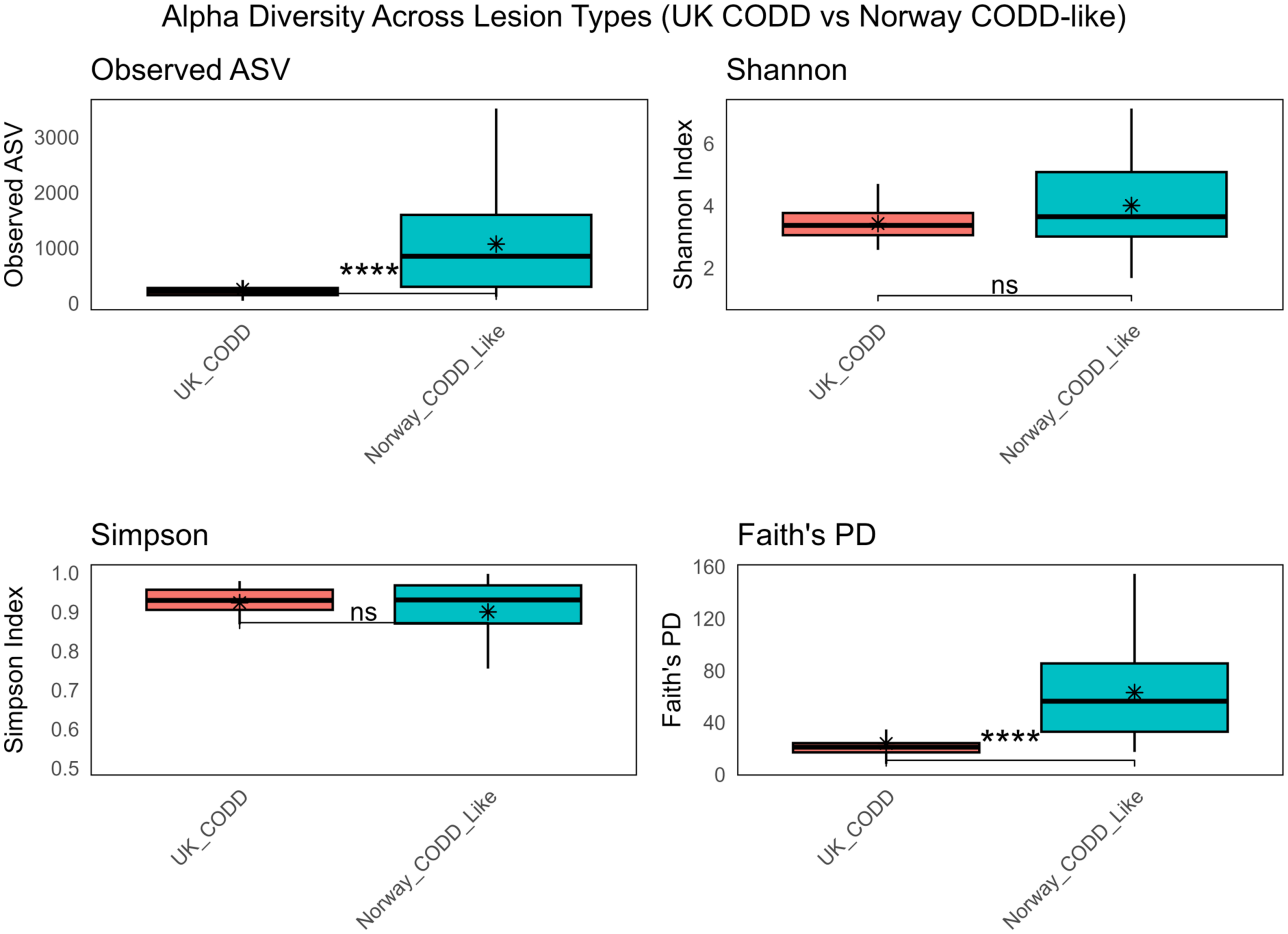
Alpha diversity box plots of UK CODD samples and Norwegian CODD-like samples. Statistical significance between the groups was evaluated using the Kruskal–Wallis test; significance levels are displayed on each panel (*** = p < 0.001, ns, not significant).

Alpha diversity analysis was used to analyse and compare within-sample bacterial diversity in terms of richness, evenness, and phylogenetic relatedness. This analysis showed that the bacterial populations present in Norwegian CODD-like lesions were significantly more diverse in terms of the number of species present (observed features, *p* < 0.001) and phylogenetic relatedness (Faith’s PD, *p* < 0.001) compared to the UK CODD lesions. However, by the Shannon diversity index (which accounts for both abundance and evenness) and the Simpson index (which reflects abundance and richness), within-sample bacterial diversity was not significantly different between the two sample types (*p* > 0.05) (Figure 3).

#### Beta diversity

**Figure 4:**
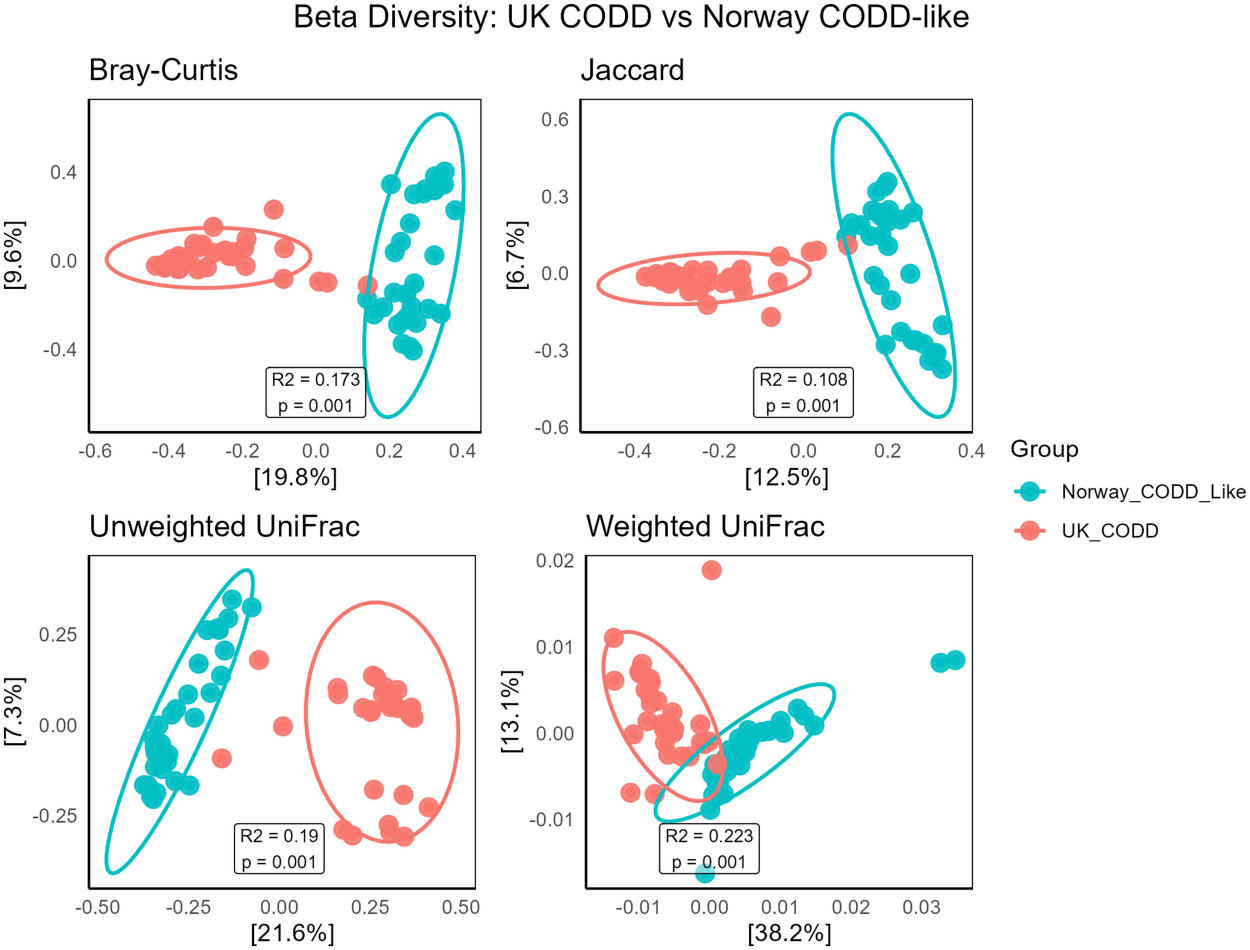
Beta diversity of Norwegian CODD-like and UK CODD samples visualised by PCoA. Points represent individual samples, and ellipses denote group dispersion. Significant differences in community composition between the groups were assessed using PERMANOVA, with *R*^2^ values indicating the effect size and *p*-values displayed on each panel.

Beta diversity analysis revealed significant differences in microbial community composition between Norwegian CODD-like and UK CODD samples across all distance metrics, including the Bray–Curtis dissimilarity (*R*^2^ = 0.173, *p* < 0.001), Jaccard index (*R*^2^ = 0.108, *p* < 0.001), unweighted UniFrac distance (*R*^2^ = 0.190, *p* < 0.001), and weighted UniFrac distance (*R*^2^ = 0.223, *p* < 0.001). The strongest effect was observed for weighted UniFrac distance, indicating that differences in dominant, phylogenetically related taxa drive separation between groups (Figure 4).

Principal component analysis identified the main bacterial genera driving separation among sample types as *Staphylococcus* spp.*, Fusobacterium* spp., *Porphyromonas* spp., *Macrococcus* spp., and *Psychrobacter* spp. (Figure 4).

#### Analysis of differentially abundant ASVs/taxa

**Figure 5:**
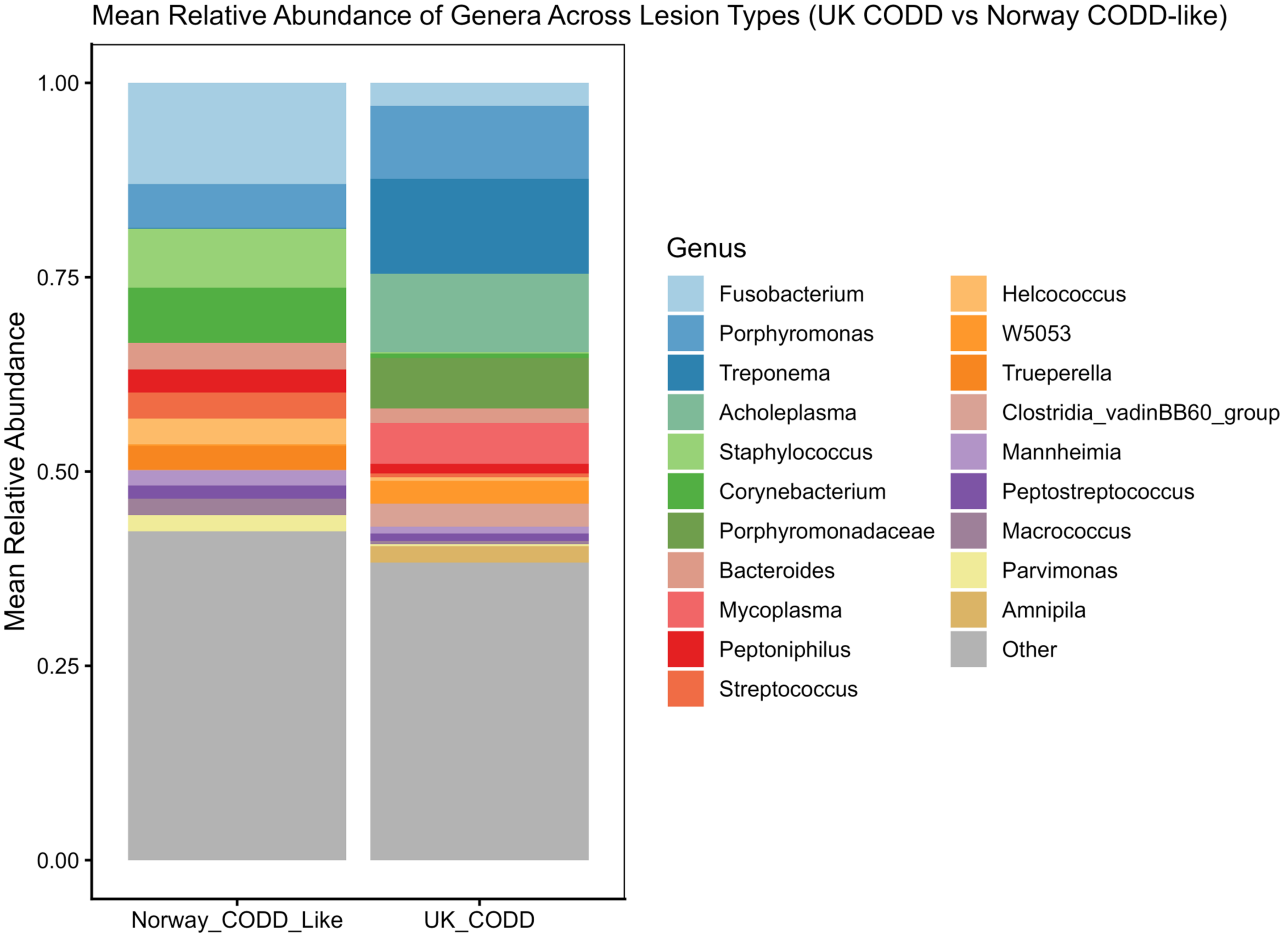
Mean relative abundance of the top 20 bacterial genera across UK CODD and Norwegian CODD-like lesion types. Remaining taxa are grouped as ‘Other’.

Visual inspection of the taxa plot of relative abundances of bacterial genera in UK CODD and Norwegian CODD-like lesions also demonstrated marked differences in bacterial genera and their abundance between the two disease categories (Figure 5).

Calculation of relative abundances of the different genera provided significant data related to the differences in microbiota composition between UK CODD and Norwegian CODD-like lesions. The UK CODD samples were generally dominated by Gram-negative anaerobic organisms, with the most abundant being *Treponema* spp., *Porphyromonas* spp. and *Acholeplasma*. The Norwegian samples comprised a mixture of Gram-positive and Gram-negative bacteria, with *Fusobacterium* spp. *Corynebacterium* spp.*, Porphyromonas* spp., and *Staphylococcus* spp. being the most abundant (Table 1).

**Table 1:** Relative abundance of the top 10 bacterial genera or families (where the genus was unclassified) in the microbiota of Norwegian CODD-like and UK CODD foot samples.

Norwegian CODD-like (*n* = 30)	Relative abundance (%)	UK CODD (*n* = 28)	Relative abundance (%)
*Fusobacterium*	13.72	*Treponema*	12.36
*Corynebacterium*	7.36	*Porphyromonas*	9.97
*Porphyromonas*	6.72	*Acholeplasma*	8.62
*Staphylococcus*	6.10	*Porphyromonadaceae*	6.33
*Helcococcus*	4.36	*Mycoplasma*	4.90
*Streptococcus*	4.29	*W5053*	3.02
*Trueperella*	4.03	*Fusobacterium*	2.96
*Peptoniphilus*	3.85	*Peptostreptococcaceae;*	2.29
*Bacteroides*	3.72	*Tissierellales uncultured*	2.20
*Macrococcus*	2.74	*Clostridia vadinBB60*	2.11

The ANCOM (36) method was used to investigate the statistical significance of differences in the relative abundance of bacterial genera among the groups. At the genus level, 74 bacterial genera were significantly differentially abundant between the two groups (Table 2).

**Table 2:** Top 10 differentially abundant genera or families (where the genus was unclassified) between Norwegian CODD-like and UK CODD samples as determined by ANCOM analysis.

Bacterial genus	W value	Sample type most abundant
*Staphylococcus*	966	Norwegian CODD-like
*Porphyromonas*	962	UK CODD
*Ezakiella*	952	UK CODD
*Lentimicrobium*	952	UK CODD
*Peptostreptococcus*	951	UK CODD
*Mycoplasma*	951	UK CODD
*Family XI*	949	UK CODD
*Peptoanaerobacter*	949	UK CODD
*Acholeplasma*	948	UK CODD
*Amnipila*	948	Norwegian CODD-like

The top 10 genera most differentially abundant between Norwegian CODD-like and UK CODD samples were *Staphylococcus, Porphyromonas, Ezakiella, Lentimicrobium, Peptostreptococcus, Mycoplasma, Family XI (genus unclassified), Peptoanaerobacter, Acholeplasma, and Amnipila. Treponema* was also significantly differentially abundant between UK CODD and Norwegian CODD-like lesions, ranked as the 23rd most differentially abundant genus (*W* = 933) and more abundant in the UK samples.

### Comparison of the microbiota associated with Norwegian foot lesions

In a further attempt to explore the bacteriological nature of the Norwegian CODD-like foot lesions observed at the Norwegian abattoir, the microbiota of the 30 CODD-like, 7 benign footrot, and 29 healthy Norwegian foot biopsy samples were compared.

#### Alpha diversity

**Figure 6:**
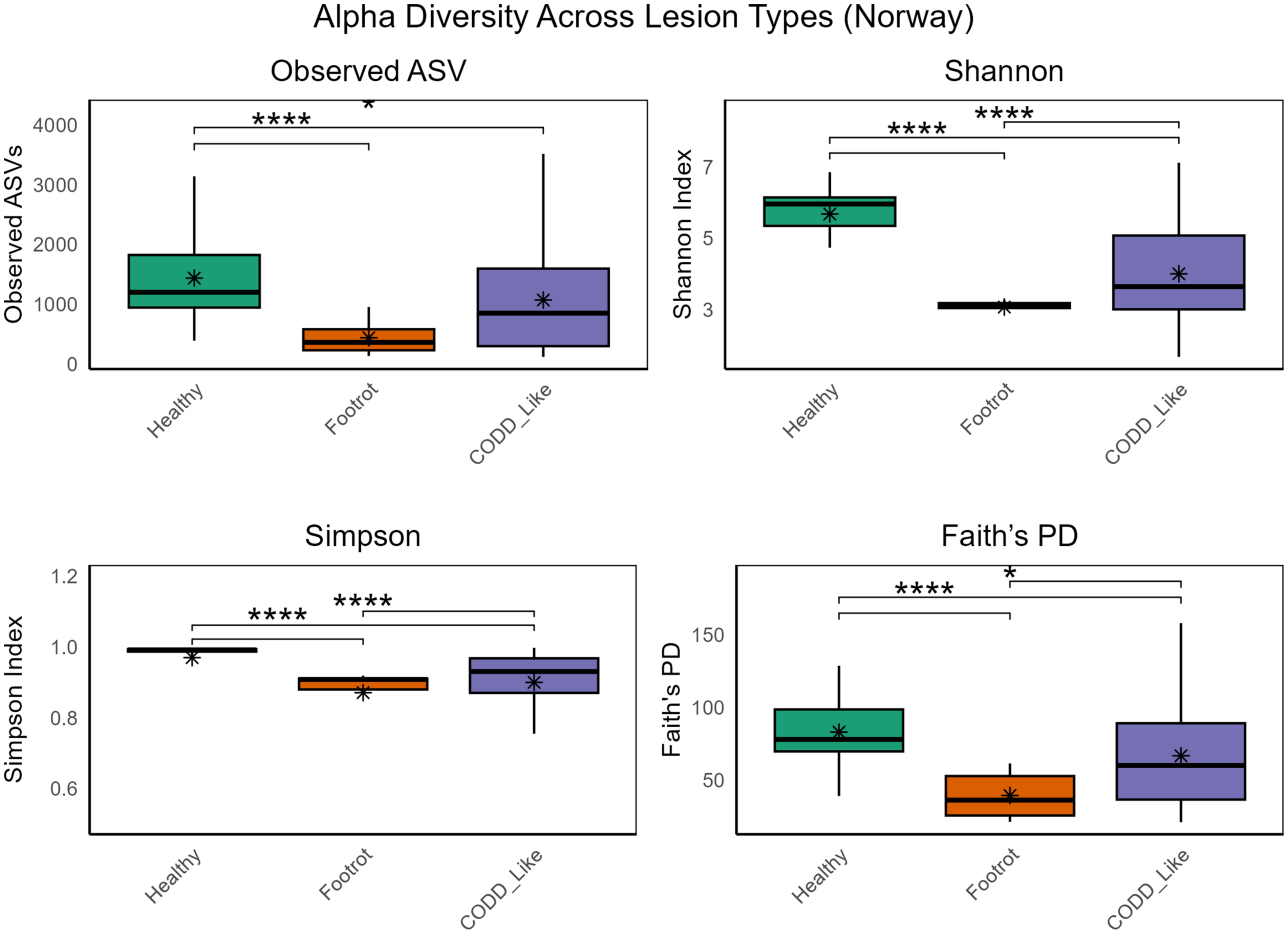
Alpha diversity box plots of Norwegian healthy, footrot, and CODD-like samples. Statistical significance between the groups was evaluated using the Kruskal–Wallis test; pairwise comparisons were performed using Wilcoxon rank-sum tests with Benjamini–Hochberg correction for multiple testing. Significance levels are displayed on each panel (* = p < 0.05, *** = p < 0.001, ns, not significant).

Pairwise comparisons revealed that both benign footrot and CODD-like lesions showed a significant reduction (*p* < 0.05) in bacterial species number, relative abundance, richness (Simpson index), evenness (Shannon diversity index), and phylogenetic diversity (Faith’s PD) compared to healthy feet. CODD-like lesions and healthy skin had similar richness (observed features; *p* > 0.05), whereas benign footrot lesions were significantly less rich than healthy feet (*p* < 0.05). No significant differences were observed between the microbiomes of footrot and CODD-like lesions in terms of alpha diversity (Figure 6).

#### Beta diversity

**Figure 7:**
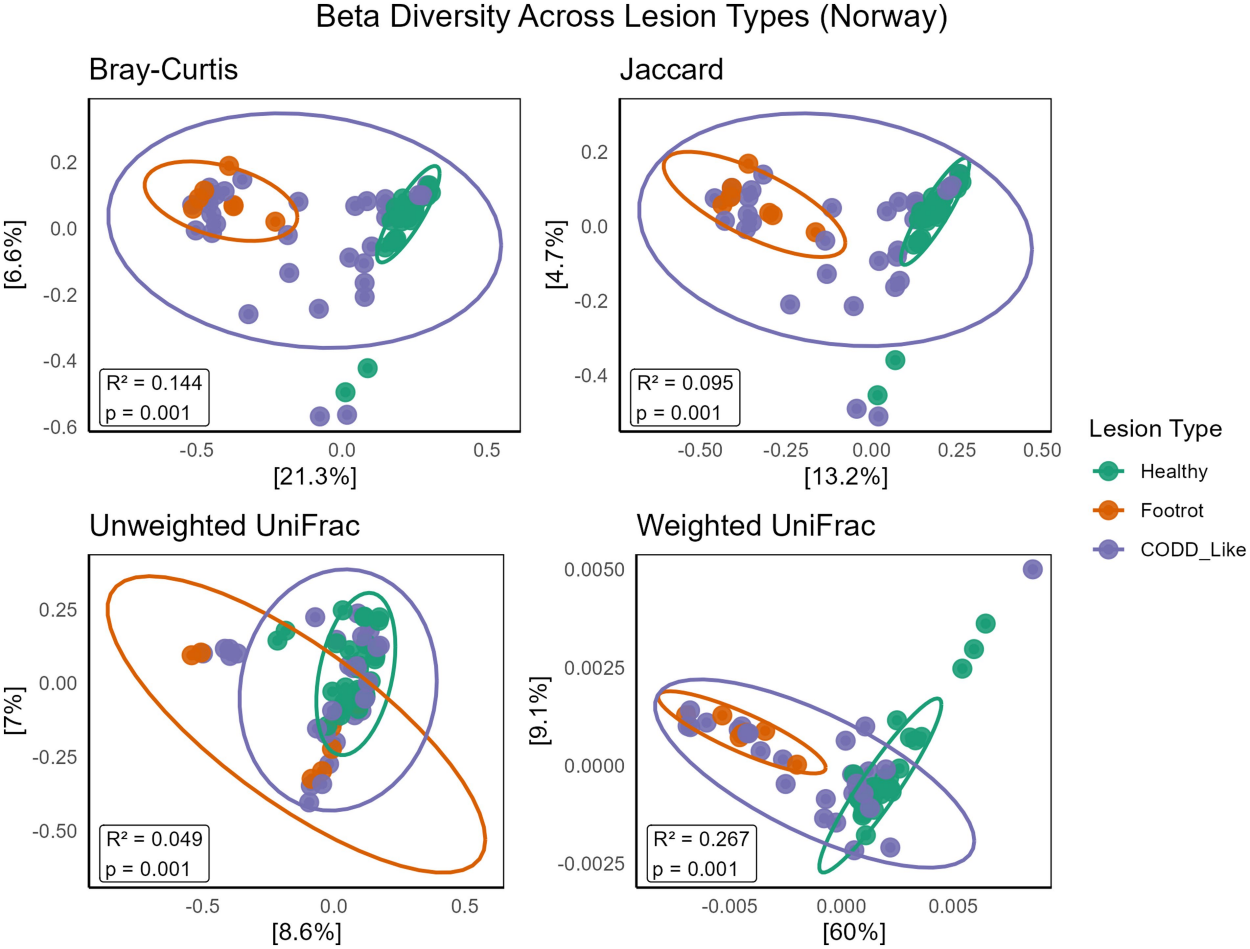
Beta diversity of Norwegian healthy, footrot, and CODD-like samples visualised by PCoA. Points represent individual samples, and ellipses denote group dispersion. Significant differences in community composition between the groups were assessed using PERMANOVA, with *R*^2^ values indicating the effect size and *p*-values displayed on each panel.

Beta diversity analysis found that bacterial community composition differed among healthy, footrot, and CODD-like samples. Weighted UniFrac distance (*R*^2^ = 0.27) indicated that much of this difference can be attributed to changes in the relative abundance of phylogenetically distinct taxa. Using principal component analysis, the top five ASVs driving separation among the samples were two *Staphylococcus* ASVs*, Fusobacterium, Porphyromonas, and Macrococcus* (Figure 7).

#### Analysis of differentially abundant ASVs/taxa among the sample groups

Taxonomic analysis was carried out at the genus level to minimise information loss due to unclassified samples at the species level.

**Figure 8:**
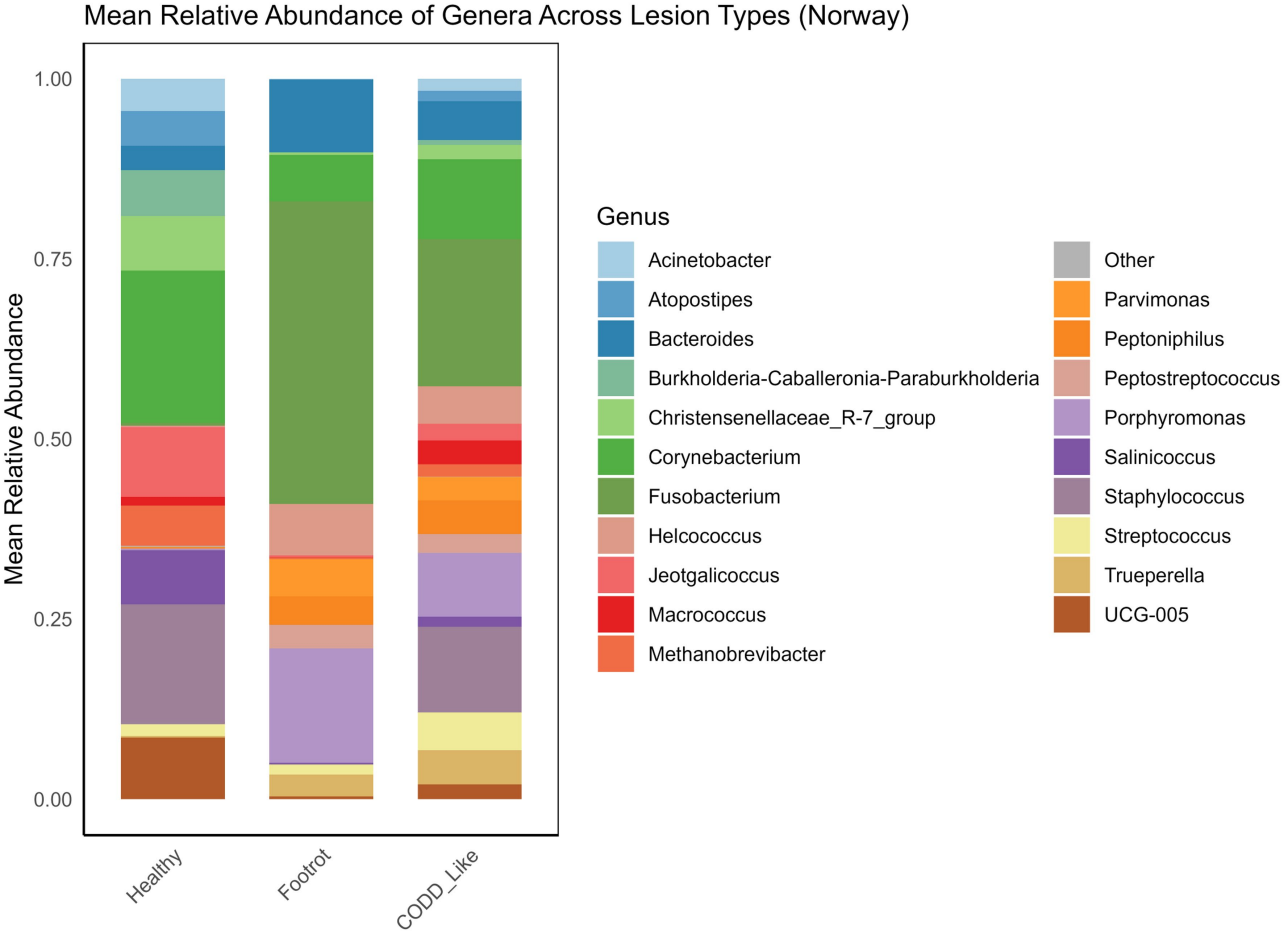
Mean relative abundance of the top 20 bacterial genera across lesion types (healthy, footrot, and CODD-like) in Norwegian samples. Remaining taxa are grouped as ‘Other’.

Visual inspection of the taxa plot supported the greater bacterial diversity in healthy feet compared to diseased feet (in particular, the benign footrot samples) and the relative importance of *Fusobacterium* and *Porphyromonas* species in both diseased foot samples (Figure 8). *Corynebacterium and Staphylococcus* were also common in both CODD-like and healthy foot samples (Table 3).

**Table 3:** Relative abundance of the top 10 bacterial genera or families (where the genus was unclassified) in the microbiota of Norwegian CODD-like, benign footrot, and healthy foot samples.

Norwegian CODD-like	Relative abundance (%)	Benign footrot	Relative abundance (%)	Healthy	Relative abundance (%)
*Fusobacterium*	12.71	*Fusobacterium*	34.44	*Corynebacterium*	7.86
*Corynebacterium*	7.87	*Porphyromonas*	11.91	*Staphylococcus*	5.23
*Porphyromonas*	6.25	*Bacteroides*	7.63	*Jeotgalicoccus*	2.96
*Staphylococcus*	5.99	*Helcococcus*	5.02	*UCG-005*	2.71
*Helcococcus*	4.05	*Corynebacterium*	4.69	*Christensenellaceae R7 group*	2.45
*Streptococcus*	3.98	*Deinococcus*	4.01	*Salinicoccus*	2.13
*Trueperella*	3.73	*Parvimonas*	3.68	*Methanobrevibacter*	1.93
*Peptoniphilus*	3.61	*Moraxellaceae*	2.93	*Pseudomonas*	1.81
*Bacteroides*	3.46	*Peptoniphilus*	2.81	*JG30-KF-CM45*	1.80
*Macrococcus*	2.54	*Peptostreptococcus*	2.51	*Lachnospiraceae*	1.56

The ANCOM (36) method was used to investigate differentially abundant bacteria among the three disease classification groups. At the genus level, ANCOM identified 12 bacterial genera as differentially abundant (W ≥ 70% of pairwise comparisons), indicating robust and consistent differences between the groups. These genera included *Fusobacterium*, *Peptiphilus*, *Parvimonas*, *Helcococcus*, *Porphyromonas*, *Trueperella*, *Murdochiella*, S5-A14a, *Peptococcus*, *Filifactor*, and *Staphylococcus* (Table 4).

**Table 4:** The 12 differentially abundant genera between Norwegian CODD-like, benign footrot, and healthy foot samples as determined by ANCOM analysis.

Bacterial genus	W	Sample type most abundant	Reject null hypothesis
*Fusobacterium*	1712	Footrot & CODD-like	TRUE
*Peptoniphilus*	1711	Footrot & CODD-like	TRUE
*Parvimonas*	1711	Footrot & CODD-like	TRUE
*Helcococcus*	1711	Footrot & CODD-like	TRUE
*Peptostreptococcus*	1711	Footrot & CODD-like	TRUE
*Porphyromonas*	1709	Footrot & CODD-like	TRUE
*Trueperella*	1704	Footrot & CODD-like	TRUE
*Murdochiella*	1,699	CODD-like	TRUE
*S5-A14a*	1,699	Footrot & CODD-like	TRUE
*Peptococcus*	1,691	CODD-like	TRUE
*Filifactor*	1,683	Footrot & CODD-like	TRUE
*Staphylococcus*	1,549	Healthy & CODD-like	TRUE

The proposed primary bacterial aetiological agents of CODD in the UK (5), *T. phagedenis, T. medium, and T. pedis,* were not identified in the Norwegian dataset. However, treponeme bacteria were mostly identified at the genus level, with only a few identified at the species level, and none were significantly differentially abundant between the groups. *Dichelobacter nodosus*, the primary aetiological agent of footrot, was identified in the Norwegian dataset. Although numerically more common in the benign footrot samples (0.1% of ASVs) compared to CODD-like (0.00008% of ASVs) and healthy samples (0.0000128%), it was not statistically significantly different.

## Discussion

Since CODD was first identified in the UK in 1997 (43), the appearance of novel skin or hoof lesions in sheep understandably raises concern among farmers and clinicians about the possible emergence of this painful and costly disease in flocks. Since CODD is known to be a polymicrobial disease (5), with no commercially available, validated laboratory diagnostic tests, alternative diagnostic approaches are needed. This study aimed to explore whether 16S rRNA sequencing of skin lesion biopsies, in combination with gross pathology, could be used to determine if novel foot lesions observed in Norwegian abattoirs were consistent with CODD.

The study clearly demonstrated that the novel Norwegian CODD-like lesions were distinct from UK CODD lesions in terms of gross pathology and microbial composition. The principal pathological differences were that the novel CODD-like lesions occurred in the skin *above* the coronary band and *below* the carpus or tarsus and none involved any separation of the hoof capsule from the underlying hoof laminae, as is typical of CODD lesions (44) (Figures 1, 2).

The microbiota of UK CODD lesions and the novel CODD-like lesions both exhibited marked dysbiosis compared to healthy foot skin samples (Figure 6) (19). In both disease states, bacterial diversity, measured by the total number and variety of microbial species, was reduced relative to healthy controls. This is a common finding in disease states, where reduced diversity may result from the overgrowth of pathogenic bacteria and/or a reduction in beneficial bacteria (45).

Despite this shared loss of bacterial diversity compared to healthy tissue, diversity analyses revealed that the microbial community composition of the two disease states (UK CODD and CODD-like lesions) was distinct. Alpha diversity analysis revealed that, although the richness and evenness of bacterial populations were broadly similar, there were significant changes in the abundance and phylogeny of the taxa present. This is reflected in the beta diversity analysis, where significant differences in microbial community composition were particularly evident in abundance-weighted metrics (the Bray–Curtis dissimilarity and weighted UniFrac distance). Examination of the relative abundance of specific taxa in each sample type and ANCOM analysis identified a notable shift towards Gram-negative taxa, as has been widely reported in other foot skin diseases of cattle (46) and sheep (47, 48). This shift was less pronounced in CODD-like lesions, where a more balanced mixture of Gram-positive and Gram-negative taxa was present (Table 1). At the genus level, the taxa identified as differentially abundant were consistent with a shift towards a dysbiotic, anaerobe-dominated microbiome associated with disease states, which was more pronounced in UK CODD lesions (45). Genera such as *Fusobacterium*, *Porphyromonas*, and *Parvimonas* are well-recognised opportunistic anaerobes associated with tissue degradation and polymicrobial infections (49). The presence of additional anaerobic genera, including *Peptoniphilus*, *Peptococcus*, and *Filifactor*, further supports the establishment of a low-oxygen, proteolytic environment within diseased tissue.

In UK CODD lesions, the most abundant genera were *Treponema, Porphyromonas, Acholeplasma, Mycoplasma,* and *Fusobacterium* (Table 1). This bacterial profile closely mirrors findings from a recent meta-analysis of bacterial populations in bovine digital dermatitis (BDD) lesions, in which *Treponema, Mycoplasma, Porphyromonas,* and *Fusobacterium* were the genera most strongly associated with the disease (46). Furthermore, recent UK studies have also highlighted strong associations between *Mycoplasma, Acholeplasma, and Treponema* in BDD, providing further evidence of the close microbiological link between bovine and ovine forms of digital dermatitis (50, 51).

Therefore, it is clear that the novel Norwegian CODD-like lesions represent a distinct disease from UK CODD. Accordingly, the bacterial populations were compared with those of biopsies obtained from benign footrot lesions (42), also collected from the Norwegian abattoirs. Diversity analysis demonstrated that the bacterial communities of the novel CODD-like lesions and benign footrot lesions were distinct from healthy skin across all diversity metrics, indicating a loss of microbial richness and/or evenness in diseased tissue. Both conditions did share several abundant genera—including *Fusobacterium*, *Corynebacterium*, *Porphyromonas*, *Helcococcus*, *Bacteroides*, *Peptoniphilus,* and *Peptostreptococcus* (Table 3)—suggesting the presence of a broadly similar, anaerobic, infection-associated microbiome. However, important differences were observed in their relative abundance, indicating that, while these conditions share a common microbial framework, they differ in community structure. CODD-like lesions were characterised by higher proportions of Gram-positive genera such as *Staphylococcus*, *Streptococcus*, *Trueperella,* and *Macrococcus*. These genera are commonly associated with early colonisation, opportunistic infection, and pyogenic disease processes (52, 53). In contrast, and consistent with previous studies of footrot microbiomes (5, 43), benign footrot lesions were more strongly associated with anaerobic genera such as *Fusobacterium* and *Porphyromonas*, which are linked to tissue degradation, proteolytic activity, and chronic infection (49).

The evidence from the current study indicates that the novel CODD-like lesions are clinically and microbiologically distinct from UK CODD and Norwegian benign footrot. A more plausible differential diagnosis includes physical trauma or ruptured foot abscesses, with subsequent invasion by common foot pathogens such as *Fusobacterium* and *Porphyromonas* (19, 47, 54). Another possibility is the recently described ovine condition, ‘ulcerative skin disease’ (16). This disease presents as apparently contagious, well-demarcated, circular ulcerative dermatitis located between the coronary band and the carpal or tarsal joints, which is similar to the observations of the novel lesions described here. Bacteriologically, ulcerative skin disease is associated with *Fusobacterium necrophorum* and *Streptococcus dysgalactiae*; interestingly, both genera were also detected in the novel CODD-like lesions described in the present study. Further investigations, including histopathology and molecular microbiology (e.g., quantitative PCR analysis), would be useful to explore this association further.

Several limitations of this study should be considered. Comparisons between different classes of foot lesions and their associated microbial communities may be influenced by variables that were not controlled for, including differences in sampling time (Norway: 2020–2022; UK: 2016), sampling location (Norwegian abattoir versus UK farms), and DNA extraction methods. Variability in sampling time and location may introduce differences in environmental microbial contamination, which could influence sequencing results.

However, superficial contamination was partially mitigated by dry wiping prior to sampling, and the key bacterial taxa identified as differing between disease states are unlikely to be explained solely by environmental contamination or methodological variation. Furthermore, previous microbiome studies have successfully compared samples collected from different environments, suggesting that the major biological signals observed here are robust (50, 51).

The Norwegian study used DNA extraction methods that are currently recommended for bacterial microbiome analysis, combining mechanical disruption, heat treatment, and chemical extraction. In contrast, the older UK method from 2016 used only heat and chemical extraction, without the additional mechanical disruption step. However, both DNA extraction kits have been used in other ruminant foot microbiome studies and, in particular, both have been able to extract treponemal bacterial DNA from tissue samples and implicate them in the corresponding disease aetiologies (19, 20, 22, 50, 55). Furthermore, the quality and quantity of DNA extracted using both methods met the quality control criteria of the same sequencing laboratory.

To minimise study variation, identical study protocols were used for lesion scoring, the same UK veterinarian scored all foot lesions, and biopsy samples were collected using the same methodology. After collection, the extracted DNA from the Norwegian samples was sent to the UK. All subsequent laboratory processes, sequencing, and bioinformatic analysis were carried out in the same UK laboratory, using the same techniques for samples from both countries.

It is also worth considering that the 30 CODD-like samples were convenience samples collected at abattoirs. As such, the study does not reflect disease prevalence at the farm level in Norway and may be subject to selection bias, as animals presented for slaughter may not be representative of the wider population.

Finally, a lower number of control (healthy) samples were collected, relative to diseased samples, which may reduce statistical power. However, consistent and statistically significant differences in both alpha and beta diversity metrics, as well as in differential abundance analyses, were detected, suggesting that the main biological signals identified are robust.

Nonetheless, the study demonstrates the potential of 16S rRNA sequencing to differentiate between foot lesion types, and, given the advent of low-cost, portable sequencing equipment, this could become a feasible diagnostic approach for polymicrobial diseases in commercial veterinary laboratories.

## Conclusion

The study used gross pathological lesion description in combination with *16S rRNA* gene sequencing of the bacterial microbiome to investigate the novel CODD-like lesions from Norwegian abattoirs. Gross pathology and bacterial diversity analysis revealed that the novel CODD-like lesions were pathologically and bacteriologically distinct from CODD. Further comparison of the microbiota with healthy and footrot samples revealed that the CODD-like lesions were dysbiotic and harboured a distinct bacterial population compared to the footrot samples. Based on their gross pathology, alternative differential diagnoses for these novel lesions include trauma, ruptured foot abscesses, or ulcerative skin disease.

## Data Availability

The original UK data sets generated and analysed during the study are available at: https://www.ncbi.nlm.nih.gov/sra/PRJNA658364 (accession number PRJNA658364). The data has previously been analysed and published here (47). The Norwegian data sets generated and analysed during the study are available at European Nucleotide Archive https://www.ebi.ac.uk/ena/browser/home (accession number PRJEB87708), Secondary accession number ERP170903.
