# Supplementary figures and images for: Application of 16S rRNA gene amplicon sequencing in the investigation of novel ovine skin lesions in Norway
Source: Front Vet Sci. 2026 Jun 3;13:1802983. doi: 10.3389/fvets.2026.1802983 (PMC13276613; doi:10.3389/fvets.2026.1802983)

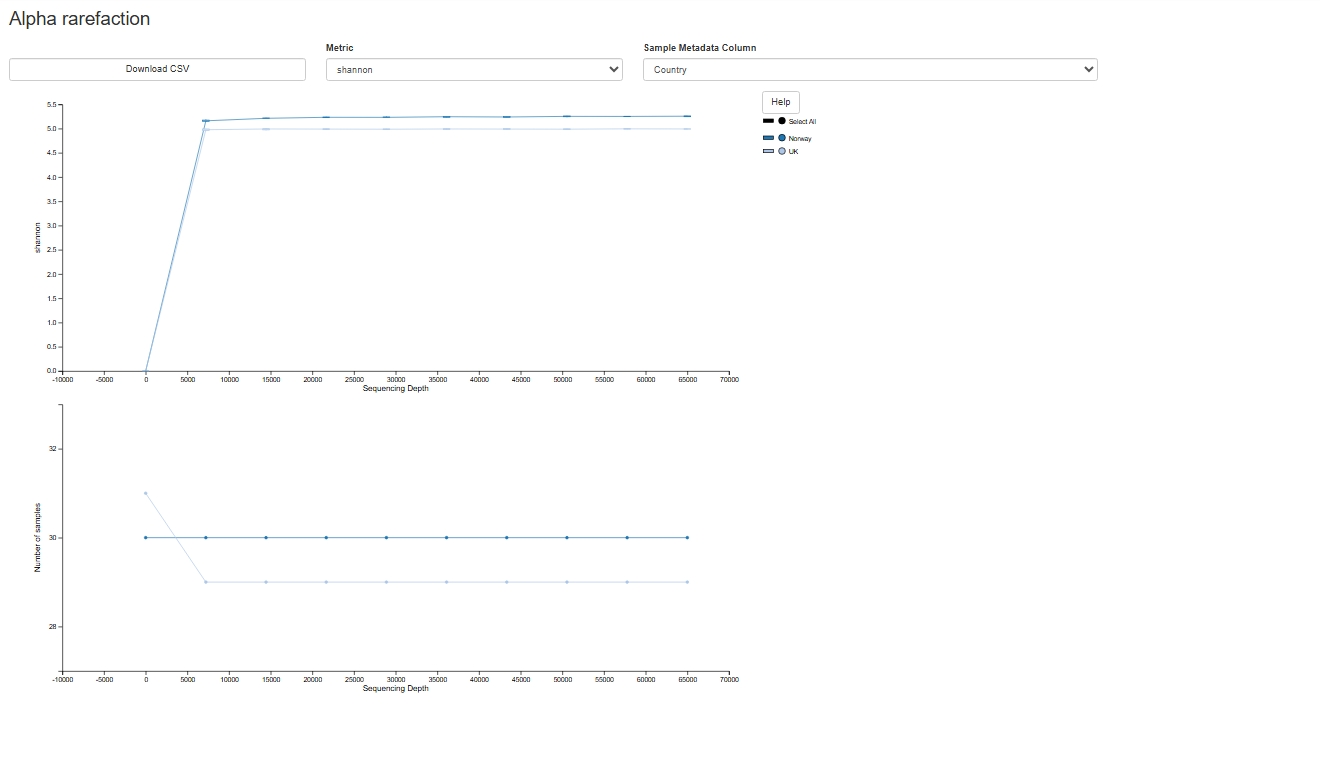

Supplement: Supplementary file 1 [file supplementary_file_1.jpeg]
